# Mandatory sex selection and mitochondrial transfer

**DOI:** 10.1111/bioe.12462

**Published:** 2018-07-06

**Authors:** Reuven Brandt

**Keywords:** assisted reproduction, mitochondria, mitochondrial donation, reproductive risk, sex selection

## Abstract

The Institute of Medicine has recently endorsed arguments put forward by John Appleby calling for mandatory sex selection against female offspring in the initial trials of mitochondrial replacement techniques. In this paper I argue that, despite this endorsement, the reasons offered by Appleby for mandatory sex selection are inadequate. I further argue that plausible revisions to Appleby's arguments still fail to convincingly defend such an intrusive policy. While I remain neutral about whether intending parents making use of mitochondrial replacement techniques ought to have access to sex selection, I conclude that to date the case for mandatory sex selection has not been satisfactorily made.

## 1 INTRODUCTION

In a historic first, the U.K. has recently approved two mitochondrial replacement procedures, namely maternal spindle transfer and pro‐nuclear transfer, for preventing the transmission of mitochondrial disease from affected women to their offspring.^1^ In the U.S.A., the Institute of Medicine (IOM)^2^ has recently released a report calling for more research into these mitochondrial replacement techniques (MRT) in order to assess their clinical potential.^3^ While there is optimism both in the U.K. and in the U.S.A. that mitochondrial replacement will prove to be a useful tool for preventing the transmission of mitochondrial disease, questions remain about its long‐term safety. Consequently, there has been much discussion about what precautions should be in place for the first human trials, especially when it comes to measures intended to protect the welfare of future generations.

Part of what makes developing appropriate safety protocols challenging in the case of mitochondrial replacement is that mitochondrial DNA is heritable, and thus downstream health effects beyond the patient, donor, and subsequent child need to be taken into account. Given that mitochondrial DNA follows a strict maternal inheritance pattern,^4^ the IOM in the U.S.A. has recommended that sex selection be mandatory in the initial trials of the procedures so that only male offspring are created.^5^ Doing so would prevent the transmission of any mitochondria‐linked complications to future generations. Once the health of the initial cohort of male offspring is assessed, the scientific community could then determine whether the procedures are safe enough to make available without the sex‐based restriction. In defence of this policy, the IOM cites corroborating arguments put forward by John Appleby, and it is these arguments that will be the primary subject of this paper.^6^

It is worth noting that the IOM's approach is not indicative of a broader consensus. The U.K.'s Human Fertilization and Embryology Authority (HFEA) has eschewed any similar form of mandatory sex selection. Their approach for preventing downstream harm following the initial implementation of the procedures is to encourage follow‐up on the initial group of children created following the procedures, and on the basis of their findings to determine whether regulations governing mitochondrial replacement techniques ought to be amended.^7^ Given that we currently lack a widely accepted framework for conducting risk/benefit assessments when evaluating procedures that may carry health risks for future generations, neither the IOM nor the HFEA take an approach that contravenes some well‐established norm. Nevertheless, I will argue that Appleby's line of argument, cited by the IOM in support of *mandated* sex selection, is flawed.

As in many debates about reproductive ethics, concerns about non‐identity arise when assessing whether future people could in fact be harmed by mitochondrial replacement. Though some have argued that the relevance of the non‐identity problem differs between the two approved techniques,^8^ the justification for sex selection is that it would prevent harm to offspring in generations further downstream than those initially created using the techniques. Consequently, the non‐identity problem remains relevant regardless of the method of mitochondrial replacement employed. In order to avoid wading into a detailed discussion of the non‐identity problem, my strategy will be to proceed under the assumption that we can cogently talk about harm done to future generations even if the individuals created are not numerically identical to those that would exist in the absence of mitochondrial replacement. I take this as a more stringent approach to addressing the arguments put forward by Appleby, as he seems to proceed with this assumption in mind. Otherwise put, by making this assumption I will be addressing Appleby's argument on his own terms with respect to non‐identity concerns. However, those who think that the non‐identity problem constrains how we apply the concept of harm to future people may think that mandatory sex selection cannot be justified by Appleby's argument because harm to downstream generations arises only in cases where subsequent individuals who inherit mitochondrial disease have lives that are not worth living.^9^

## 2 THE ARGUMENT FOR MANDATORY SEX SELECTION

In their report on mitochondrial replacement techniques, the IOM defends a policy of initial mandatory sex selection against female offspring on the grounds that this restriction would reduce the risk to future generations.^10^ This recommendation is based in part on work done by Annelien Bredenoord et al., who argue that the prevention of harm to future generations ought to fall within the scope of medically justifiable reasons for sex selection,^11^ and in part by work done by Appleby, who argues that, in the case of mitochondrial donation trials, such medically justified sex selection ought to be mandatory while the risks associated with the procedures are being studied.^12^ The basic argument for sex selection put forward by Appleby relies on two distinct steps:

(a) establishing that, in the case of mitochondrial replacement, selecting against female offspring is justifiable on medical grounds; and

(b)   establishing that such medical sex selection ought to be mandatory, at least initially.

Assessing what evidence would be required for (a) rests to a large extent on one's views of the permissibility of sex selection more generally. Those who hold that sex selection is a *pro tanto* significant wrong would have a high threshold for what medical concerns ought to outweigh any presumptive prohibition against sex selection,^13^ while those who take a more liberal stance on the permissiveness of sex selection may think that no medical justification is necessary for sex selection at all.^14^ However, regardless of one's view on the permissibility of sex selection – medical or otherwise – step (b) requires considerable justification as it seems to be a *prima facie* infringement on individuals' reproductive autonomy. Consequently, even if someone thinks that sex selection ought to be permissible in the case of early recipients of mitochondrial replacement, the claim that sex selection ought to be *mandatory* in such cases requires additional argument. Because (b) is controversial regardless of what one thinks about (a), I will focus on showing that the justification for (b) presented by Appleby is inadequate. This strategy allows us to focus specifically on justifying mandatory sex selection.

### 2.1 The argument from harm minimization

The principal argument given by Appleby, and one echoed by the IOM, for mandating sex selection in the initial trials of mitochondrial donation is that such a policy would reduce the risk to future generations by preventing the downstream transmission of potentially pathological mitochondrial DNA.^15^ To this end, Appleby states, ‘because the mitochondrial genome is maternally inherited, clinicians applying to the HFEA for a license to use MRTs should be required to use sex selection techniques to select for male offspring (whenever possible) … in order to reduce mtDNA‐related transgenerational health risks.^16^ However, this move is all too quick. If we are bound by an indefeasible obligation to use only those interventions that carry the lowest risk to future generations then we ought not permit experimental use of mitochondrial replacement in the first place. This is because alternatives that have a near‐zero risk of transmitting pathological mtDNA already exist – donor‐assisted reproduction and adoption.^17^ By endorsing trials of mitochondrial replacement at all, Appleby already tacitly accepts that certain goods for intending parents, such as having genetically related offspring, can justify exposing future children to some degree of avoidable risk. This leaves open the possibility that reproductive autonomy conceived in a manner that excludes mandatory sex selection may also be a good that justifies exposing future children to some degree of avoidable risk.

While Appleby does not explicitly discuss what goods for prospective parents might justify putting future children at risk, he does claim that a policy of mandatory sex selection at the research stage is unlikely to harm society, future children, or prospective parents. It therefore seems to follow that mandating sex selection is ethically permissible, because doing so would reduce risks to future generations without bringing about any harm. However, Appleby's argument dismissing mandatory sex selection as harmful to prospective parents is problematic for two reasons. First, it rests on an account of harm that is utterly implausible. Second, when taken in concert with his commitment to harm minimization, it leads to the conclusion that female carriers of mitochondrial disease should be prohibited from reproducing with their own gametes in *all* cases, including reproduction via mitochondrial replacement.

Let us consider more closely Appleby's claim that mandatory sex selection would not result in harm to intending parents. On this subject he states, ‘considering how few prospective parents would likely be granted clinical access to MRTs (the chances are that it would be <10 cases each year), it is hard to imagine how bringing only males into existence would…cause suffering to the prospective parents.’^18^ Put more generally, Appleby's view seems to be that a policy that imposes burdens on members of some group X cannot be said to cause harm to members of X, if X is small. The glaring problem here is that Appleby assesses the potential for harm to prospective parents solely by considering the number of individuals affected rather than the nature of the burdens that such a policy might produce. This is an ethical approach that, upon slight reflection, could license all manner of serious injustices. But even if we grant Appleby this contentious view, it still only undermines his argument. Consider that prohibiting all carriers of mitochondrial disease from reproducing with their own gametes regardless of method of conception (unassisted sexual reproduction, mitochondrial replacement, or otherwise) would be the most effective method for preventing the transmission of mitochondrial disease to future generations. Furthermore, given that relatively few individuals are both carriers of mitochondrial disease and committed to using their own gametes to reproduce despite the possible risks to their offspring, such a prohibition would likely affect only a relatively small number of individuals.^19^ By Appleby's own lights then, such an intrusive and burdensome policy would be perfectly morally acceptable: because the number of people affected would be small, on Appleby's account no harms would arise. Taken in concert with his stated commitment to harm minimization, Appleby's account of harm thus in fact requires him to endorse *prohibiting* carriers of mitochondrial disease from reproducing with their own gametes in *all* circumstances. This is clearly incompatible with his endorsement of mitochondrial replacement trials.

That some individuals may have an obligation to refrain from using their own gametes when reproducing is not a view that ought to be dismissed outright. There are indeed positions in the literature that suggest that certain reproducers may have a duty to use donor gametes. For instance, Bennett and Holm have recently argued that the ‘own biological children’ proviso in Savulescu and Kahane's defence of the principle of procreative beneficence^20^ is untenable, and that the principle of procreative beneficence may sometimes require individuals to seek out third‐party gametes.^21^ Appleby's argument suggests a similar conclusion at least in the cases involving pathological mitochondrial DNA, and this is incompatible with permitting trials into mitochondrial replacement regardless of whether sex selection is implemented.

## 3 APPLEBY'S ARGUMENT RECAST

### 3.1 The pragmatic argument

There are, however, ways in which one might defend a position similar to Appleby's. One might think that in theory it would indeed be preferable if those at risk of transmitting mitochondrial disease would refrain from reproducing with their own gametes in all circumstances, but think that enforcing a policy of this kind would be politically, ethically, and pragmatically untenable. After all, such a prohibition would, among other things, require a mechanism for preventing individuals from engaging in unassisted reproduction using their own gametes. Furthermore, one could also recognize that some individuals are committed to having genetically related offspring to such an extent that they will continue to attempt to do so even if their own previous attempts exemplify the consequences of producing children with severe mitochondrial disease.^22^ Because these individuals cannot be prevented from reproducing in a risky manner, one might conclude that the best way to minimize harm is to permit such individuals' participation in trials of mitochondrial replacement while also enforcing a policy of mandatory sex selection. Doing so would at least reduce the risk of creating offspring with mitochondrial disease, and eliminate the possibility of its re‐emergence in future generations. Call this the *pragmatic harm reduction* argument.

The pragmatic harm reduction argument rests on the key premise that trials into mitochondrial replacement procedures are likely to leave the immediate offspring and future generations better off, or at least no worse off, than if the trials were not conducted. Appleby's assessment of the possible risks to offspring following use of the approved procedures supports the plausibility of this premise. While some have argued that novel negative health outcomes may arise as a consequence of incompatibility between donor mitochondria and the nuclear DNA of the developing zygote, Appleby notes that it is unclear whether such risks are any greater following mitochondrial replacement than in other forms of human reproduction.^23^ The concern that Appleby offers as justification for mandatory sex selection is that mitochondrial replacement therapies might not be completely effective at eliminating all of the pathological mitochondria from affected ova. Consequently, there is risk that offspring might harbour small amounts of pathological mitochondrial DNA. Even if this remnant pathological mitochondrial DNA does not result in mitochondrial disease in the immediate offspring, it is possible that mitochondrial disease may re‐emerge in generations further downstream – a possibility that is supported by animal models.^24^ A policy of mandatory sex selection against female offspring while such risks are being assessed would prevent the possibility of the downstream re‐emergence of mitochondrial disease.

At first blush, recasting Appleby's proposal as a pragmatic means for minimizing harm to offspring may seem to provide solid footing for mandatory sex selection. Current research suggests that participants in the trial are much less likely to have offspring with mitochondrial disease, and sex selection will prevent mitochondrial disease from occurring in future generations in cases where the procedure does not expunge all pathological mitochondrial DNA. However, even this modified version of Appleby's argument cannot justify mandating sex selection for all potential trial participants. In order to see why, imagine a carrier of mitochondrial disease who is both intent on creating a genetically related child and who would be a perfect candidate for the trial, but for her staunch objection to sex selection. Note that such an objection might not stem from a strict preference for having a female child, which we might find morally suspect. Instead, the individual might simply object to having the sex of her child ‘artificially’ determined, or may object to a practice that she thinks contributes to and/or normalizes morally suspect non‐medical sex selection.^25^ Excluding this individual from the trial will not result in a reduction of overall harm, and may indeed result in greater harm to offspring. This is because if this individual reproduces outside of a mitochondrial replacement trial there is a much higher likelihood that resultant offspring will have mitochondrial disease. Furthermore, any female offspring created will be at higher risk of carrying levels of pathological mitochondria sufficient to create offspring with mitochondrial disease even if they themselves present as asymptomatic. With respect to Appleby's principal concern, downstream transmission of mitochondrial disease, so long as mitochondrial replacement trials do not risk increasing the amount of pathological mitochondrial DNA likely to be present in offspring (and so far no one has suggested that such a risk exists), participation in the trial poses less of a risk than unaided conception. The pragmatic risk minimization argument therefore does not support mandatory sex selection in all cases because in some circumstances denying an individual access to the trial will result in a greater risk to subsequent offspring.

A possible response may be that if sex selection is made voluntary, individuals who would have agreed to participate in a trial that mandated sex selection may choose to decline this additional risk‐reducing measure. Thus while removing the sex‐selection requirement may open participation in the trial to those with stark objections to selection against female offspring, it may also result in some individuals making riskier reproductive decisions than they would if presented with the choice between unassisted reproduction and participation in a mitochondrial replacement trial with mandatory sex selection. For instance, an individual who would opt for MRT with mandated sex selection over unassisted reproduction might choose MRT without sex selection if given the choice, despite the transgenerational risks. Call this the *risky choice objection*. This worry is certainly reasonable, though whether it justifies mandating sex selection is a complex empirical question that rests on determining whether such a policy would result in more risk‐averse decision‐making overall, taking into consideration any transgenerational harms that may arise. Though this objection is not one that can be fully addressed a priori, it is worth noting that the risky choice objection cuts both ways. Similar worries arise with respect to permitting trials into mitochondrial donation in the first place. Consider that the most effective way for female carriers to avoid transmitting mitochondrial disease to future generations is to forgo reproducing with their own gametes, even when sex selection is taken into consideration. One could thus similarly argue that while permitting trials into mitochondrial replacement might reduce harm in certain instances by providing a less risky option for those carriers determined to reproduce using their own gametes, it may also encourage individuals who would have otherwise opted to become parents without using their own gametes, or perhaps even forgone parenthood altogether, to partake in the trials despite the greater risks this poses to potential offspring. In the absence of empirical evidence to settle the question, an appeal to the risky choice objection cannot warrant mandatory sex selection without also bringing into question the permissibility trials into mitochondrial replacement techniques.

### 3.2 Balancing of interests argument

A second way one might defend Appleby's policy recommendation is to forgo his controversial account of harm and accept that carriers of mitochondrial disease can be harmed by having their reproductive choices limited – both by policies that affect their reproductive decisions and by mitochondrial disease. Adopting such a position gives rise to a tension between the interests of intending parents and the interests of future children. As noted previously, what is best for the wellbeing of future children is preventing carriers of mitochondrial disease from reproducing with their own gametes, but enforcing such a policy would limit reproductive choice by precluding carriers from having genetic offspring. Adopting a policy of mandatory sex selection until the safety and effectiveness of mitochondrial replacement is established might seem like the appropriate middle‐ground between the competing interests of future offspring and intending parents. Though this approach is not the most risk‐averse for future generations, if the procedure fails to fully prevent the transmission of pathological mitochondrial DNA the effects will not be transmitted to future generations. And while mandatory sex selection would diminish reproductive autonomy to an extent, it still allows intending parents to have genetically related offspring, which is very important to many intending parents.

That a policy of mandatory sex selection correctly balances these competing interests would of course not be uncontroversial. Those who place little normative weight on the preference for rearing genetically related offspring,^26^ endorse an account of reproductive autonomy that is narrow in scope,^27^ or place more weight on the interests of offspring than on those of intending parents^28^ might reject the permissibility of trials into mitochondrial replacement given the risks. Conversely, those who are convinced by non‐identity type arguments,^29^ endorse an expansive account of reproductive autonomy, or think that current research demonstrates that mitochondrial replacement is safe (enough) might find mandatory sex selection indefensible, even when restricted to the initial cohort. However, apart from disagreement about how to properly balance the competing concerns of intending parents and future offspring, there is an additional and more theoretical reason to be suspicious that this approach could justify mandatory sex selection. As will be explained in what follows, this justification for mandatory sex selection restricts reproductive autonomy on the grounds that the *offspring* of trial participants may choose to reproduce in a risky manner. Given that all reproduction results in offspring who may themselves reproduce in a risky manner, the argument in its present form does not justify mandatory sex selection.

As a reminder, the goal of mitochondrial replacement is to replace all of an ovum's native mitochondria with healthy donor mitochondria. However, the process is not perfect, and small amounts of pathological mitochondrial DNA variants may become incorporated into the resultant zygote. As noted previously, recent evidence suggests that there is a potential risk that the presence of small amounts of pathological mitochondrial DNA could result in illness in offspring, and that pathological mitochondrial DNA may accumulate in the female germline, posing a risk to future generations.^30^ Broadly speaking then, there are three possibilities that may arise as a consequence of inadvertent carryover following mitochondrial replacement. The first is that no clinically relevant carryover occurs at all. In other words, no offspring suffer from mitochondrial disease and the risk that mitochondrial disease will re‐emerge in subsequent generations is negligible. The second is that a small amount of mitochondrial carryover results in asymptomatic carriers – female offspring who themselves do not suffer from mitochondrial disease, but who risk passing mitochondrial disease on to subsequent generations. Lastly, individuals could suffer from mitochondrial disease and any female offspring created would be at risk of passing the disease on to subsequent generations.

If it turns out that mitochondrial replacement is likely to result in one of the aforementioned undesirable outcomes, sex selection will have no impact on the development of mitochondrial disease in the first generation of offspring. Mitochondrial disease has the potential to arise in both female and male offspring. If the level of pathological mitochondrial DNA remains sufficiently low following the procedure, neither male nor female offspring are likely to develop mitochondrial disease. What sex selection would prevent is the possibility of female symptomatic and asymptomatic carriers transmitting mitochondrial disease to their offspring. However, if approaches such as the one endorsed by the HFEA are implemented, transmission of mitochondrial disease is likely to arise only if affected female offspring *choose* to reproduce despite knowing the risks. This is because their trials will involve long‐term follow‐up of offspring in order to determine the effectiveness and safety of the treatment,^31^ and individuals seeking MRT will be encouraged to inform female offspring created using the techniques about the circumstances of their conception so that they can make informed decisions about reproducing later in life.^32^ Consequently, first‐generation female offspring would in all likelihood be aware that they risked transmitting mitochondrial disease to their offspring. Such awareness would be most acute in cases where offspring develop mitochondrial disease prior to reaching reproductive age, if mitochondrial replacement proves to be ineffective in some cases. However, even in cases where female offspring develop late‐onset mitochondrial disease or are asymptomatic carriers, we can still expect that first‐generation female offspring will be aware that they may risk transmitting mitochondrial disease. Most optimistically, follow‐up tests might be able to quantify the risk of transmission in particular individuals. But even in the absence of individualized risk assessments, the follow‐up will alert first‐generation offspring to the fact that they may have inherited pathological mitochondrial DNA. Risk‐avoidance measures, such as using mitochondrial replacement (assuming the procedure is found to be effective) or donor ova could be employed by female offspring who choose to reproduce.

The purpose of sex selection is to eliminate the possibility of creating individuals who might opt to reproduce without taking measures to reduce the likelihood of transmitting mitochondrial disease. Otherwise put, it is a policy that restricts the reproductive autonomy of a class of individuals on the grounds that *their offspring* may fail to take appropriate precautions if they choose to reproduce. This is a weak ground for such an intrusive policy. For one, in all cases of reproduction there is a risk that one's offspring may reproduce in a manner that is likely to cause harm to their offspring. Progenitors generally do not have the ability to prevent their offspring from exposing themselves to mutagens or teratogens prior to conceiving or while gestating, nor can they generally prevent their offspring from reproducing while suffering from severe infectious diseases that could be transmitted to their offspring. If procreators had a duty to reproduce in a manner that ensures their offspring do not make risky reproductive decisions, procreators would be required to exercise much more control over their adult offspring than is currently considered acceptable, or else only create infertile offspring. At a minimum, in order to show that harm to future individuals outweighs reproductive autonomy in the case of mitochondrial replacement trials, proponents of mandatory sex selection would have to show that the risk to future offspring posed by allowing female offspring to be created as part of the trial is likely to be significantly greater than the likelihood of risky reproduction that is currently accepted.^33^

A possible response may be that there is some important difference between the risks posed by pathological genetic material and other sources of reproductive harm. For instance, we might point to the fact that genetically inheritable sources of harm are generally more transgenerationally persistent than other kinds of inheritable harm, or that the harms they pose are unique in the extent to which they are difficult to avoid. While much can be said about the normative importance of different kinds of inheritable risk factors, one reason to be suspicious of the claim that the risks posed by trials into mitochondrial replacement justify a restriction as severe and demanding as state‐mandated sex selection is that similar restrictions are not imposed in other cases where there is a risk of transmitting genetic abnormalities.

Consider an individual who is intent on reproducing and is a carrier of a severe recessive genetic disorder, such as beta‐thalassemia. That individual can avoid creating a child who will suffer from the disease in at least three permissible ways: using donor gametes in place of his/her own, not reproducing with an individual who is also a carrier of the disease, or reproducing with a carrier but using in vitro fertilization (IVF) and pre‐implementation diagnosis (PGD) in order to select against homozygous offspring who will suffer from the disease. Note, however, that only the first option, employing donor gametes, eliminates the possibility of creating an offspring who might be an asymptomatic carrier.^34^ This risk is not trivial. Individuals using one of the other two methods have a 50% chance of creating a child who is an asymptomatic carrier. If these offspring do not themselves take precautions when reproducing, they may create children with severe genetic diseases, or produce another generation of asymptomatic carriers. However, despite the risk to future generations, there is currently no requirement in either the U.K. or the U.S.A. for individuals with recessive genetic abnormalities to reproduce in a manner that prevents the creation of asymptomatic carriers. In fact, in the U.K. access to IVF and PGD as a means of preventing the transmission of genetic disease is indicated only in cases where reproducers risk creating a child who will suffer from the disease.^35^ This is not to suggest that it would be unethical for individuals to make use of reproductive interventions in order to avoid creating asymptomatic carriers,^36^ but it does show that restricting reproductive autonomy in order to prevent the creation of offspring who could choose to reproduce in a manner that may transmit a genetic disease is highly unusual. Of course, individuals sympathetic to negative eugenics might think that such state‐mandated limitations on reproductive autonomy is warranted, and would similarly take no issue with Appleby's policy recommendation. However, such a position would be quite the departure from established norms, and as such would require a much more robust defence than simply stating that it best prevents the transmission of mitochondrial disease. After all, there are many circumstances in which limiting reproductive autonomy would prevent the possibility of genetic diseases emerging in subsequent generations. A proponent of state‐mandated sex selection would at least have to acknowledge and accept the wider implications entailed by the argument advanced in its favour, or else provide reasons for why similar restrictions on reproductive autonomy are not warranted for the prevention of other genetic disease.

While I cannot evaluate all the reasons someone might have for treating trials into MRT as a special case, one that might come to mind is that different norms apply in the context of clinical trials. A full response to this proposal would require in‐depth engagement with the literature on research ethics that space does not allow. However, the following hypothetical case illustrates why we should be skeptical that the research context justifies restriction on autonomy of the kind endorsed by the IOM. Consider the following hypothetical example.

Scientists are working on two new flu vaccines, vaccine A and vaccine B, designed for a sub‐population for whom the standard vaccine is normally ineffective. Vaccine A uses a new kind of adjuvant to stimulate the immune response to the flu virus, but scientists are not certain that it will result in protection equivalent to that of traditional adjuvants, and there are suspicions that it may even leave individuals as susceptible to the flu as those not immunized at all. By contrast, vaccine B is derived from an influenza strain that has been genetically modified to be particularly virulent, then attenuated so that it poses little health risk. However, researchers are not certain that the attenuation process used in vaccine B is perfectly effective. Consequently, vaccine B poses a small risk of infecting people with a genetically engineered ‘super flu’. Here vaccine B results in the potential for a novel harm caused by the experimental treatment itself, while vaccine A risks leaving individuals as vulnerable as they would be if they had not participated in the trial at all.

Given that flu shots are not mandatory in most contexts, this distinction has implications for what restrictions on autonomy are reasonable for those participating in the studies of the two vaccines. For instance, while public health officials recommend that individuals receive the flu vaccine as a means of protecting vulnerable populations, individuals are still free to use public transit, attend public gatherings etc. even if they choose not to get a flu vaccine. Consequently, it would be problematic if, as a condition of participating in the trial of vaccine A, the state prohibited participants from using public transit or attending crowded public events. This is because even if vaccine A fails, it still results in no greater to risk to vulnerable populations than already exists. Such a restriction would have the perverse effect of demanding a higher commitment to public health measures from those choosing to enter into a trial for an effective vaccine than from those who make no such effort. The same is not true of individuals involved in the trial of vaccine B, because it risks exposing others to a greater degree of risk (a more virulent form of the flu) than they would otherwise face. Hence, policies restricting the activities of individuals involved in the study of vaccine B may be justified on the grounds that doing so protects the public from a novel risk created by the trials. As has been noted, Appleby's worry is that mitochondrial replacement might not remove all pathological mitochondrial DNA, leaving open the possibility that mitochondrial disease may not be completely eliminated from the germline. Given that it is permissible for those with mitochondrial disease to reproduce, trials into mitochondrial replacement are more like trials into vaccine A than they are like trials into vaccine B. Endorsing mandated sex selection would thus mean endorsing the placement of greater restrictions on the trail participants who are taking steps to avoid transmitting mitochondrial disease than on those who choose to eschew any harm‐reduction measures.

If, however, the procedure leads to new sex‐linked transmissible risks that are greater than those present in non‐controversial forms of reproduction then there may be a case for imposing sex selection. While haplotype incompatibility is a possible contender highlighted by the IOM, as noted previously there is evidence that MRT will not put offspring at greater risk of serious health consequences arising from incompatibility between mitochondrial DNA and nuclear DNA. The IOM also lists epigenetic effects and as yet unforeseen complications as risks to take into consideration. However, both could affect the nuclear genome in addition to the mitochondrial genome and so could be transmissible via either sex.^37^ Appeals to these potential worries thus do not serve as a justification for sex selection.

## 4 Conclusion

While this paper focuses on the regulation of mitochondrial replacement techniques, the lessons learned can be generalized to other similar cases. Recall that a major problem with Appleby's argument is that he thinks that it is ‘hard to imagine’ how prospective parents could be severely adversely impacted by a policy mandating sex selection on the grounds that only a few individuals would be affected by the policy. Apart from resting on a view of harm that is implausible, when taken in concert with the view that we ought to minimize overall harm, this approach undermines the permissibility of trials into mitochondrial donation. This is because if we do not have to take into consideration the adverse impact on would‐be reproducers, it is unclear why, at least at the theoretical level, it would not be preferable to simply prohibit those who risk transmitting diseases from reproducing with their own gametes. What this discussion shows is that when determining how to regulate interventions directed at preventing the transmission of diseases from intending parents to generations downstream, we cannot simply appeal to the interests of future children.

Once we acknowledge that the debate requires more nuance, matters quickly become murky, as exemplified in the discussion of other possible defences of mandatory sex selection. It is not immediately clear how to balance reproductive autonomy against the possibility of harm to downstream generations. Part of what makes such determinations difficult is that all reproducers run the risk of having offspring who may choose to reproduce in a risky fashion. While current practice might be mistaken, it seems to eschew restricting reproductive autonomy on the grounds that one's offspring may choose to act in a way that brings about harm, even in cases where their offspring might be asymptomatic carriers of serious genetic disorders. And while those who endorse adopting a more restrictive approach to reproductive autonomy might be tempted to endorse policies like Appleby's on pragmatic grounds, without some empirical data indicating how reproducers are likely to respond when access to potentially beneficial technological advancements requires ceding reproductive autonomy, it is unclear whether such policies will in fact result in more risk‐averse reproduction.

To be clear, I do not think that the arguments presented here establish that mandatory sex selection in the case of mitochondrial replacement trials is impermissible. Doing so would require, among other things, a conclusive refutation of negative eugenics. While much work has been done on this topic, I have not delved into it in any depth here. What I have shown is that a proper ethical evaluation of policies that interfere with reproductive autonomy is much more complex than simply identifying the course of action that appears to pose the least risk to future generations. Making clear the complexity involved in evaluating such policies is important not just for academic reasons, but also because organizations, such as the IOM, look to applied ethics when faced with novel questions about regulation. Regardless of one's view of mandatory sex selection in mitochondrial replacement trials, it should be clear that the arguments relied upon by Appleby do not support this conclusion.

Finally, my analysis has been restricted to assessing the permissibility of *state‐mandated* sex selection in mitochondrial replacement trials. Questions about where the limits of medically justifiable sex selection lie, or whether reproducers ever have a duty to make use of it, while pressing, lie outside the scope of this paper.

## Conflict of interest

The author declares no conflict of interest.

## Footnotes

1
The act, The Human Fertilisation and Embryology (Mitochondrial Donation) Regulations 2015 No. 572, came into force on October 29, 2015. The Human Fertilization and Embryology Authority approved the first license for use of mitochondrial replacement techniques to Newcastle University in March 2017.

2
The Institute of Medicine has subsequently been renamed the National Academy of Medicine.

3
Committee on the Ethical and Social Policy Considerations of Novel Techniques for Prevention of Maternal Transmission of Mitochondrial DNA Diseases. (2016). *Mitochondrial replacement techniques: Ethical, social, and policy considerations*. Washington, DC: National Academies Press. 27054230

4
Mishra, P., & Chan, D. (2014) Mitochondrial dynamics and inheritance during cell division, development and disease. *Nature Reviews Molecular Cell Biology*, *15*(10), 634–646. 10.1038/nrm3877PMC425004425237825

5
Op. cit. note 3, p. 120.

6
Ibid.

7
Human Fertilization and Embryology Authority. (2017). *Code of practice* (8th ed.). London, UK: Human Fertilization and Embryology Authority, p. 290.

8
Wrigley, A., Wilkinson, S., & Appleby, J. B. (2015). Mitochondrial replacement:ethics and identity. *Bioethics, 29*(9), 631–638. 10.1111/bioe.12187PMC462070326481204

9
For a defence of this kind of view, see Heyd, D. (2009). The intractability of the nonidentity problem. In M. Roberts & D. Wasserman (Eds.), *Harming future persons*:*Ethics, genetics and the nonidentity problem* (pp. 3–25). New York, NY: Springer. For a good discussion of the non‐identity problem, see Boonin, D. (2014). *The non‐identity problem and the ethics of future people* Oxford, U.K.: Oxford University Press and Parfit, D. (2017). Future people, the non‐identity problem, and person‐affecting principles. *Philosophy and Public Affairs, 45*(23), 118–157.

10
Op. cit. note 3. p.120.

11
Bredenoord, A. L., Dondorp, W., Pennings, G., & De Wert, G. (2010). Avoiding transgenerational risks of mitochondrial DNA disorders: A morally acceptable reason for sex selection? *Human Reproduction, 25*(6), 1354–1360. 10.1093/humrep/deq07720364025

12
Appleby, J. B. (2015). The ethical challenges of the clinical introduction of mitochondrial replacement techniques. *Medicine, Health Care and Philosophy*, *18*(4), 501–514. 10.1007/s11019-015-9656-3PMC459119926239841

13
Note that U.K. law permits sex selection only when it is to prevent serious sex‐linked illnesses. See the Human Fertilization and Embryology Act 2008, Schedule 2.

14
For example, see Wilkinson, S. (2010). *Choosing tomorrow's children: The ethics of selective reproduction*. Oxford, U.K.: Oxford University Press, Chapter 8.

15
The IOM lists the potential for haplotype incompatibility as an additional reason for sex selection. However, Appleby notes that it remains unclear whether mitochondrial replacement results in a risk of haplotype incompatibility that is greater than in any other form of human reproduction. Appleby, op. cit. note 12, p. 509. Appleby does not appeal to haplotype incompatibility in his argument for mandatory sex selection.

16
Ibid. note 9, p. 510.

17
Baylis, F. (2013). The ethics of creating children with three genetic parents. *Reproductive Biomedicine Online*, *26*(6), 531–534. 10.1016/j.rbmo.2013.03.00623608245

18
In the U.K., pathological mutations in mitochondrial DNA occur at a rate of 2.9 per 100,000 individuals: see Gorman, G. S., Schaefer, A. M., Ng, Y., Gomez, N., Blakely, E. L., Alston, C. L., … Taylor, R. W. (2015). Prevalence of nuclear and mitochondrial DNA mutations related to adult mitochondrial disease. *Annals of neurology*, *77*(5), 753–759. Also note that only female carriers risk transmitting the disease, and that some would employ donor gametes, adopt, or forgo reproducing. 10.1002/ana.24362PMC473712125652200

19
Ibid.

20
See footnote 3 in Savulescu, J., & Kahane, G. (2009). The moral obligation to create children with the best chance of the best life. *Bioethics*, *23*(5), 274–290. 10.1111/j.1467-8519.2008.00687.x19076124

21
Note that Holm and Bennett consider this conclusion implausible, and thus see it as a reason for rejecting the principle of procreative beneficence: Holm, S., & Bennett, R. (2014). The proper scope of the principle of procreative beneficence revisited. *Monash Bioethics Review*, *32*(1–2), 22–32. 10.1007/s40592-014-0003-x25434062

22
For an example, see the story of Sharon Bernardi as reported by the BBC. Banerji, R. (2012, September 20). The woman who lost all seven children. *BBC World Service* Retrieved from http://www.bbc.com/news/magazine-19648992.

23
Op. cit. note 12, p. 506.

24
Yamada, M., Emmanuele, V., Sanchez‐Quintero, M. J., Sun, B., Lallos, G., Paull, D., …Hirano, M. (2016). Genetic drift can compromise mitochondrial replacement by nuclear transfer in human oocytes. *Cell Stem Cell*, *18*(6), 749–754. 10.1016/j.stem.2016.04.001PMC932649827212703

25
For reasons why someone may hold this view, see de Melo‐Martn, I. (2013). Sex selection and the procreative liberty framework. *Kennedy Institute of Ethics Journal*, *23*(1), 1–18. 10.1353/ken.2013.000023678627

26
For example, Haslanger S. Family, ancestry and self: What is the moral significance of biological ties. *Adoption and Culture, 2*, 91–122.

27
Benatar, D. (2006). Reproductive freedom and risk. *Human Reproduction*, *21*(10), 2491–2493. 10.1093/humrep/del20916775155

28
Hammond, J. (2010). Genetic engineering to avoid genetic neglect: From chance to responsibility*. Bioethics*, *24*(4), 160–169. 10.1111/j.1467-8519.2008.00685.x20394109

29
Op. cit. note 9.

30
Op. cit. note 24.

31
While follow‐up cannot be required of participants, the HFEA requires clinics to have procedures in place to conduct such follow‐up, and requires clinics to inform patients of the benefits of participating in the follow‐up. Code of Practice op. cit. note 7. Sections 33.33 and 33.34.

32

*Code of Practice* op. cit. note 7. Sections 33.20 and 33.21.

33
Though someone may argue that reproducers have an obligation to refrain from creating offspring who would have to go to great lengths to reproduce in a manner that does not pose great risk to their offspring, as will be discussed below, in other cases of genetic disease we do not normally impose this requirement on intending parents.

34
IVF and PGD could be used to screen out symptomatic carriers (heterogeneous carriers), though this is not required in either the US or the UK. As note below, this may not even be permissible in the UK.

35
Human Fertilisation and Embryology (HFE) Act 1990. *Schedule II*, Section 1ZA (2).

36
For a more detailed discussion, see De Wert, G., Dondorp, W., Shenfield, F., Devroey, P., Tarlatzis, B., Barri, P., … Pennings, G. (2014). ESHRE task force on ethics and Law22: Preimplantation genetic diagnosis. *Human Reproduction, 29*(8), 1610–1617. 10.1093/humrep/deu13224927929

37
For discussion of the potential epigenetic effects of assisted reproduction, see Van Montfoort, A. P. A., Hanssen, L. L. P., De Sutter, P., Viville, S., Geraedts, J. P. M., & De Boer, P. (2012). Assisted reproduction treatment and epigenetic inheritance. *Human Reproduction Update, 18*(2), 171–197. 10.1093/humupd/dmr047PMC328257422267841

